# Inhibition of TRPC6 reduces non-small cell lung cancer cell proliferation and invasion

**DOI:** 10.18632/oncotarget.14034

**Published:** 2016-12-20

**Authors:** Li-Li Yang, Bing-Chen Liu, Xiao-Yu Lu, Yan Yan, Yu-Jia Zhai, Qing Bao, Paul W. Doetsch, Xingming Deng, Tiffany L. Thai, Abdel A. Alli, Douglas C. Eaton, Bao-Zhong Shen, He-Ping Ma

**Affiliations:** ^1^ Department of Radiology, The Fourth Affiliated Hospital of Harbin Medical University, Harbin, Heilongjiang, China; ^2^ Molecular Imaging Research Center of Harbin Medical University, Harbin, Heilongjiang, China; ^3^ Department of Physiology, Emory University School of Medicine, Atlanta, Georgia, USA; ^4^ Department of Radiation Oncology, and Biochemistry and Winship Cancer Institute of Emory University, Atlanta, Georgia, USA; ^5^ Center for Cell and Molecular Signaling, Emory University School of Medicine, Atlanta, Georgia, USA

**Keywords:** anti-cancer drugs, cell cycle, metastasis, intracellular calcium, confocal microscopy

## Abstract

Recent studies indicate that the transient receptor potential canonical 6 (TRPC6) channel is highly expressed in several types of cancer cells. However, it remains unclear whether TRPC6 contributes to the malignancy of human non-small cell lung cancer (NSCLC). We used a human NSCLC A549 cell line as a model and found that pharmacological blockade or molecular knockdown of TRPC6 channel inhibited A549 cell proliferation by arresting cell cycle at the S-G2M phase and caused a significant portion of cells detached and rounded-up, but did not induce any types of cell death. Western blot and cell cycle analysis show that the detached round cells at the S-G2M phase expressed more TRPC6 than the still attached polygon cells at the G1 phase. Patch-clamp data also show that TRPC whole-cell currents in the detached cells were significantly higher than in the still attached cells. Inhibition of Ca^2+^-permeable TRPC6 channels significantly reduced intracellular Ca^2+^ in A549 cells. Interestingly, either blockade or knockdown of TRPC6 strongly reduced the invasion of this NSCLC cell line and decreased the expression of an adherent protein, fibronectin, and a tight junction protein, zonula occluden protein-1 (ZO-1). These data suggest that TRPC6-mediated elevation of intracellular Ca^2+^ stimulates NSCLC cell proliferation by promoting cell cycle progression and that inhibition of TRPC6 attenuates cell proliferation and invasion. Therefore, further *in vivo* studies may lead to a consideration of using a specific TRPC6 blocker as a complement to treat NSCLC.

## INTRODUCTION

Lung cancer is the most frequent tumor in the world and represents the leading cause of cancer-related deaths worldwide [1]. Non-small cell lung cancers (NSCLC) have a rather unpredictable prognosis and account for about 80% of primary lung cancers. Approximately 85% of diagnoses are made when the tumor can no longer be removed surgically, and the median survival for these people is 13 months due to a lack of effective therapies [2]. In most cases, metastasis rather than the primary tumor accounts for the major mortality of these patients. Therefore, efficacious chemotherapeutic regimens to prevent metastasis have become urgent need for the treatment of lung cancers, especially NSCLC. Recent studies suggest that intracellular Ca^2+^ plays an important role in cancer metastasis [3]. The transient receptor potential (TRP) channel is a superfamily of non-selective, Ca^2+^-permeable cation channels that is expressed in almost all mammalian cells [4, 5]. The TRP canonical 6 (TRPC6) channel is highly expressed in several types of cancer cells [6–12] including NSCLC cells [13]. Specifically, TRPC6 plays an important role in regulating not only the proliferation of hepatoma cells [14], ovarian cancer cells [12], prostate cancer cells [15], breast cancer cells [16], and NSCLC cells [13], but also the metastasis of glioblastoma [6] and head and neck squamous cell carcinomas [17]. It has also been shown that inhibition of TRPC6 channels causes the cell cycle of oesophageal cancer [8], glioma [7], and renal carcinoma cells [10] arrested at the G2/M phase. However, it remains unclear whether TRPC6 is critical for cell cycle progression and metastasis of lung cancer cells.

The A549 cell line is a good model representing highly metastatic lung adenocarcinoma cells [18]. Therefore, in the present study we used A549 cells to determine the mechanism by which TRPC6 regulates NSCLC cell proliferation and invasiveness. For the first time, we show that the expression of TRPC6 is cell cycle-dependent; we also show that specific inhibition of TRPC6 expression with siRNA against TRPC6 inhibits A549 cell proliferation by causing the cells arrested at the S-G2/M phase and attenuates their ability of invasion possibly by reducing the expression of an adherent molecule, fibronectin, and a tight junction protein, zonula occluden protein-1 (ZO-1). Since A549 is insensitive to gefitinib [19], a drug which is extensively used for treatment of lung cancers, this study may facilitate the search for alternative drugs to complement lung cancer treatment.

## RESULTS

### SKF-96365 inhibits human NSCLC A549 cell proliferation by blocking TRPC6

**Figure 1:**
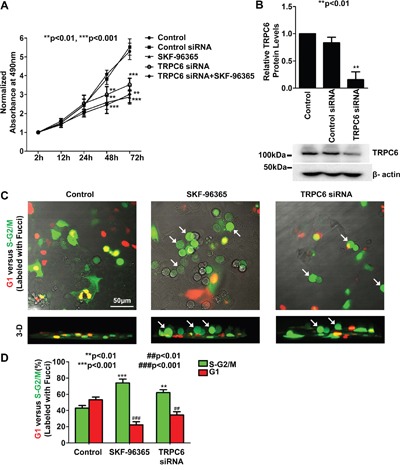
Either pharmacological block of TRPC6 with SKF-96365 or knockdown of TRPC6 expression with TRPC6 siRNA suppresses human non-small cell lung cancer A549 cell proliferation and causes A549 cells arrested at the S-G_2_/M phase **A.** Time course of MTS assay of cell proliferation. In all the experiments through the study, otherwise indicated, A549 cells were either under control conditions or transiently transfected with either 80 nM control siRNA or 80 nM TRPC6 siRNA, or treated with 5 μM SKF-96365 for 24 h, respectively. **B.** Summary plots of relative TRPC6 protein levels from three separate Western blot experiments from control cells or cells transfected with either control siRNA or TRPC6 siRNA. β-actin in the same loading membrane was immunoprecipiated with antibody to β-actin and used to normalize TRPC6 protein levels. Representative data are shown below the summary plots. **C.** Representative confocal microscopy XY plane (upper) and 3-dimentional (3-D, lower) images of cell cycle analysis with Fucci. Cells at the G_1_ phase are shown in red whereas cells at the S or G_2_/M phase are shown in green. Cells were treated for 24 h with 5 μM SKF-96365 or transfected with TRPC6 siRNA. White arrows indicate detached round cells. **D.** Summary plots of G_1_ phase (red bars) versus S-G_2_/M phase (green bars) after each treatment.

To determine whether TRPC6 blockade affects NSCLC cell proliferation, TRPC6 channels in A549 cells were either pharmacologically blocked with 5 μM SKF-96365 or knocked down with TRPC6 siRNA. MTS assays were performed to evaluate cell proliferation (Figure 1A). The data showed that treatment of A549 cells with SKF-96365 or transfection with TRPC6 siRNA resulted in a significant time-dependent reduction in cell proliferation. Transfection with control siRNA did not affect cell proliferation. SKF-96365 does not specifically block TRPC6 [20]. To eliminate the possibility that SKF-96365 inhibits A549 cell proliferation by blocking other types of Ca channels, the cells were co-treated with both SKF-96365 and TRPC6 siRNA. The data showed that co-treatment did not suppress proliferation more significantly than TRPC6 knockdown alone. To determine TRPC6 knockdown efficiency, Western blot experiments were carried out, showing that TRPC6 protein expression was significantly attenuated (Figure 1B) after transfection of TRPC6 siRNA, but not control siRNA. These data suggest that SKF-96365 inhibits A549 cell proliferation by blocking TRPC6.

### Inhibition of TRPC6 causes A549 cells arrested at the G2/M phase

Recent studies have shown that in human oesophageal cancer [8] and glioma cells [7], inhibition of TRPC6 induces cell cycle arrested at the G2/M phase. To test whether inhibition of TRPC6 reduces A549 cell proliferation by affecting cell cycle, A549 cells were transfected with a cell cycle probe, Fucci. Both SKF-96365 and TRPC6 siRNA caused cell cycle arrested at the S-G2/M phase (Figure 1C and 1D). As indicated by white arrows in Figure 1C, we noticed that both SKF-96365 and TRPC6 siRNA caused the cells rounded-up and almost detached (see 3-D images). The percentage of cells in the S-G2/M phase was increased from 43% ± 3% (control level) to 74% ± 5% (after SKF-96365; n=4, p<0.001) or increased to 62% ± 4% (after TRPC6 siRNA; n=4, p<0.01), as shown in Figure 1D (green bars). Conversely, the percentage of cells at the G1 phase (red bars) was decreased from 53% ± 3% (control level) to 22% ± 4% (after SKF-96365; n=4, p<0.001) or decreased to 35% ± 4% (after TRPC6 siRNA; n=4, p<0.01). These data suggest that either blockade of TRPC6 activity or specific knockdown of TRPC6 expression attenuates A549 cell proliferation by inhibiting cell cycle progression.

### A549 cells at the S-G2/M phase express more functional TRPC6 than those at the G1 phase

**Figure 2:**
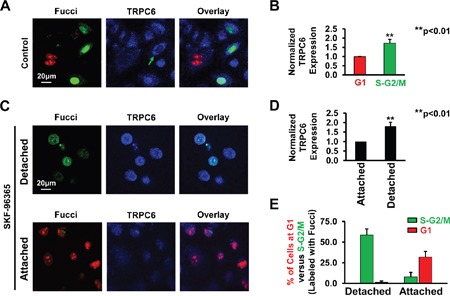
Cell cycle-dependent expression of TRPC6 **A.** Double labeling of cell cycle (red: G1 phase; green: S-G2M phase) and TRPC6 (blue) in control cells, SKF-induced detached cells, or still attached cells even after treatment with SKF. **B.** Summary plots of normalized fluorescence intensity representing TRPC6 expression levels in control A549 cells at the G1 or S-G2/M phase. **C.** Representative confocal images of detached and still attached cells after treatment for 24 h with 5 μM SKF-96365. **D.** Summary plots of normalized TRPC6 expression in detached or still attached A549 cells after treatment with SKF-96365. **E.** Summary plots of G_1_ phase (red bars) versus S-G_2_/M phase (green bars) in either detached or still attached A549 cells. These images represent data from four separate experiments showing consistent results.

**Figure 3:**
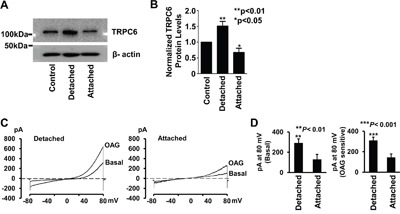
Cells responded to SKF-96365 (detached cells) express more TRPC6 than those insensitive to SKF 96365 (still attached cells) **A.** Representative Western blot of TRPC6 from control cells (untreated cells; left), SKF-induced detached cells (a population of floating cells after treatment with SKF; middle), or still attached cells (another population of cells which is still attached after treatment with SKF; right). **B.** TRPC6 protein levels were normalized with β-actin and summarized from three separate Western blot experiments. **C.** Representative whole-cell currents either from a SKF-96365-induced detached cell or from a cell which was still attached even after treatment with SKF-96365. A voltage-ramp protocol for −80 mV to 80 mV at a holding potential of −40 mV was given every minute. After recording basal current, cells were exposed 100 μM OAG to activate TRPC6 channels. **D.** Summary plots of basal and OAG-sensitive currents at 80 mV. OAG-sensitive currents were achieved by subtracting basal current from the current at 5 min after activated by 100 μM OAG.

To determine whether cell cycle-dependent expression of TRPC6 accounts for the fact that inhibition of TRPC6 caused A549 cells arrested specifically at the S-G2/M phase, but not at the G1 phase, A549 cells were double-labeled with both Fucci and TRPC6 antibody. The data show that TRPC6 levels in A549 cells at the S-G2/M phase were significantly higher than those in the cells at the G1 phase (Figure 2A and 2B). To further demonstrate this cell cycle-dependent expression of TRPC6, we also used the cells after treatment with SKF-96365. The data show that highly expressed TRPC6 at the S-G2/M phase accounts for the fact that SKF-96365 caused a subpopulation of cells, not all the cells, rounded-up and almost detached (Figure 2C–2E). To confirm the cell cycle-dependent expression of TRPC6, Western blot experiments were performed using cell lysates from control A549 cells, SKF-96365-induced detached round cells, and still attached cells after treatment with SKF-96365. The data show that the detached round cells at the S-G2/M phase express more TRPC6 than those still attached cells at the G1 phase (Figure 3A and 3B). To test whether the highly expressed TRPC6 in the round cells is functional, whole-cell patch-clamp experiments were performed. Prior to the experiments, SKF-96365 was washed out to release TRPC6 from the blocked state. Since TRPC6 is directly activated by the membrane-permeable diacylglycerol analog, 1-oleoyl-2-acetyl-sn-glycerol (OAG) [21], OAG-sensitive whole-cell currents were used to evaluate TRPC6 activity. The data show that both basal and OAG-sensitive currents in the round cells at the S-G2/M phase were significantly higher than those in the attached cell at the G1 phase (Figure 3C and 3D). These data suggest that cell cycle-dependent expression of TRPC6 accounts for the S-G2/M arrest induced by SKF-96365 in A549 cells.

### Inhibition of TRPC6 causes A549 cells detached and rounded-up by reducing intracellular Ca^2+^

**Figure 4:**
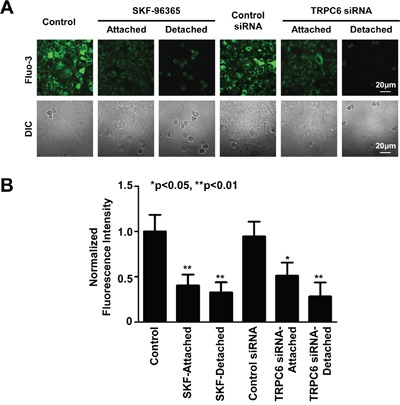
Inhibition of TRPC6 reduces intracellular Ca^2+^ **A.** Representative confocal microscopy fluorescent and DIC images of control A549 cells, still attached cells even after treatment with SKF-96365, or SKF-96365- and TRPC6 siRNA-induced detached cells. **B.** Summary plots of relative fluorescence intensity after each treatment. Data were from four separate experiments; all shows similar results. The fluorescence intensity in each treatment was normalized according to the mean of fluorescence intensity under control conditions which was set to 100%.

**Figure 5:**
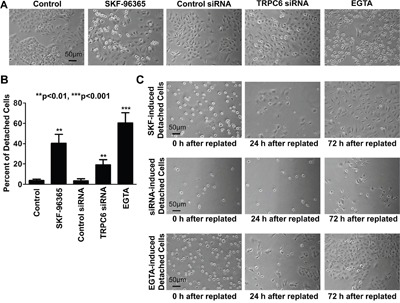
Block of TRPC6, knockdown of TRPC6 expression, or chelation of Ca^2+^ causes A549 cells rounded up and even detached **A.** Representative images of A549 cells under control conditions or treated either for 24 h with 5 μM SKF-96365, control siRNA, or TRPC6 siRNA or for 6 h with 1 mM EGTA. **B.** Summary plots of the percentage of round cells after each treatment. **C.** Representative images showing that detached cells grew back to form cell colonies at 24 h and then further proliferate at 72 h after replated in normal culture medium without any experimental manipulation. Detached cells (induced by SKF-96365, TRPC6 siRNA, or EGTA) were collected by gently blowing off with a pipette, centrifuged, stained with trypan blue to confirm the dead cells are less than 5%, and then replated into new petri dishes. After incubated for 0, 24, or 72 h in regular medium, these cells were examined under a microscope. Images represent data from four separate experiments showing consistent results.

Since TRPC6 is a Ca^2+^-permeable channel [21], theoretically, TRPC6 should modulate the levels of intracellular Ca^2+^. Therefore, intracellular Ca^2+^ was examined with Fluo-3, a Ca^2+^ probe. The data show that intracellular Ca^2+^ was significantly reduced in the cells treated with either SKF-96365 or TRPC6 siRNA, particularly in the detached cells (Figure 4A and 4B). To determine whether the reduction of intracellular Ca^2+^ accounts for the detachment, we artificially reduced intracellular Ca^2+^ by application of EGTA to the culture medium. We show that EGTA mimicked the effect of SKF-96365 and TRPC6 siRNA (Figure 5A and 5B). The percentage of detached round cells was increased, from low control levels (4% ± 1%) to 40% ± 9% after treatment with SKF-96365 (n=4; p<0.01), to 19% ± 5% after transfection with TRPC6 siRNA (n=4; p<0.01), or to 60% ± 10% after treatment with EGTA (n=4; p<0.001). The detached cells were tested with trypan blue and showed only less 5% cells were dead in each condition. In contrast, treatment with control siRNA did not cause any detachment (3% ± 2%, n=4; p>0.05). To further compare the effects of SKF-96365, TRPC6 siRNA, and EGTA on cell adhesion, the detached cells were collected and replated into new petri dishes containing regular medium. Consistent with the results from trypan blue staining, almost all the detached cells after treatment with SKF-96365 and EGTA became re-attached within a few hours, grew into cell colonies within 24 h and further proliferated with 72 h after the replating. In contrast, the cells treated with TRPC6 siRNA were still detached until at 72 h after the replating, indicating a slow recovery from TRPC6 knockdown (Figure 5C). These data suggest that inhibition of TRPC6 only causes A549 cell detachment, but does not induce any types of cell death.

### Inhibition of TRPC6 reduces invasive ability of A549 cells and decreases fibronectin and ZO-1 expression

**Figure 6:**
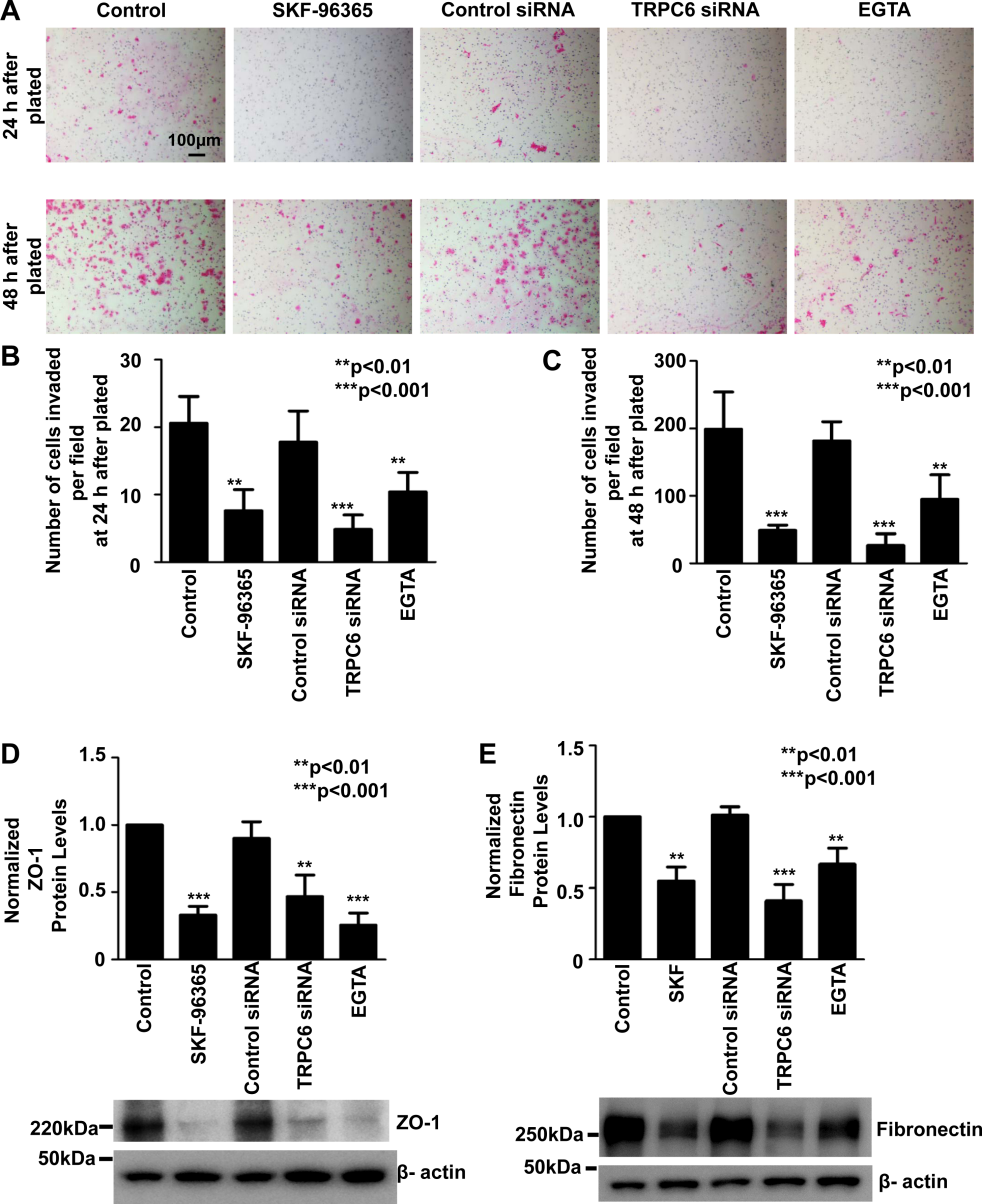
Inhibition of TRPC6 reduces A549 cell invasion and decreases expression of both ZO-1 and fibronectin, probably by reducing intracellular Ca^2+^ **A.** Representative images of cells passed through the membrane, shown in purple. A549 cells were under control conditions or treated for 24 h or 48 h with 5 μM SKF-96365, control siRNA, or TRPC6 siRNA, or for 6 h with 1 mM EGTA. Summary plots of the number of invasive cells under each condition for 24 h **B.** or 48 h **C.** per field (counted using 15 microscopy fields from three separate experiments). **D** and **E.** show normalized protein levels of either ZO-1 or fibronectin in A549 cells from three separate Western blot experiments, using β-actin as a loading control. Representative Western blot data are shown below the summary plots.

Recent studies suggest that TRPC6 [6, 9] and intracellular Ca^2+^ [3] play an important role in tumor metastasis. Therefore, the role of TRPC6 in regulating cell invasion was determined using an established method as reported previously [6]. As depicted in Figure 6A, a reduction of intracellular Ca^2+^, no matter whether it was caused by blocking TRPC6 with SKF-96365, knocking down TRPC6 with siRNA, or caused by directly chelating Ca^2+^ by EGTA, significantly attenuated A549 cell invasion. At 24 h after treatment with SKF-96365, TRPC6 siRNA, or EGTA, the number of cells per microscopy field passed through the *Transwell* membrane was reduced, from 21±4 to 8±3 (SKF-96365; *P*<0.01), 5±2 (TRPC6 siRNA; *P*<0.001), or 10±3 (EGTA; *P*<0.01), respectively (Figure 6B). The reduction in the number of cells should not result from a possible effect on cell proliferation, because neither SKF-96365 nor TRPC6 siRNA affected cell proliferation within 24 h. At 48 h after treatment with SKF-96365, TRPC6 siRNA, or EGTA, the number of cells per microscopy field passed through the *Transwell* membrane was reduced, from 199±55 to 49±8 (SKF-96365; *P*<0.001), 27±17 (TRPC6 siRNA; *P*<0.001), or 95±36 (EGTA; *P*<0.01), respectively (Figure 6C). Previous studies suggest that fibronectin is an adhesion molecule which is associated with malignant cell invasion [22] whereas ZO-1 is a tight junction protein which is also associated with cancer cell migration and invasion [23]. Therefore, Western blot experiments were performed to determine whether inhibition of TRPC6 affects the expression of fibronectin and ZO-1. The data show that treatment of A549 cells with SKF-96365, TRPC6 siRNA, or EGTA significantly decreased the protein levels of fibronectin and ZO-1. In contrast, control siRNA did not induce any effects (Figure 6D and 6E). Taken together, these data suggest that inhibition of TRPC6 attenuates the invasive ability of A549 cells probably by decreasing fibronectin and ZO-1 expression.

## DISCUSSION

The TRP superfamily consists of several subfamilies; most of them are involved in cell proliferation, metastasis, and apoptosis [24, 25]. It has been argued that different TRP channels play different roles in prostate cancer, either promoting cell proliferation or inducing apoptosis [26]. However, they all are Ca^2+^-permeable channels [4, 5] which should modulate the levels of intracellular Ca^2+^, even though one type of channel could produce opposite effects. For example, enhanced TRPC6 channel activity accounts for malignant cell proliferation [12–16, 27]; conversely, excess activation of TRPC6 induces apoptosis in glomerular podocytes [28, 29]. Therefore, as we argued in our recent publication [27], we suggest that moderate elevation of intracellular Ca^2+^ should promote cell proliferation whereas severe elevation of intracellular Ca^2+^ should initiate apoptosis.

In the present study, we show that inhibition of TRPC6 decreases intracellular Ca^2+^ and attenuates the proliferation of human NSCLC A549 cells by causing cell cycle arrested at the S-G2/M phase. It is not surprising that intracellular Ca^2+^ provides important regulatory signals during the cell cycle [30]. However, for the first time, we show that TRPC6 modulates intracellular Ca^2+^ in A549 lung cancer cells and therefore controls cell cycle progression and that TRPC6 is highly expressed in NSCLC cells at the S-G2/M phase rather than at the G1 phase. Although it has long been noticed that the expression of some channels such as Ca^2+^-activated K^+^ channels, voltage-dependent K^+^ channels, and volume-sensitive Cl^−^ channels is cell cycle-dependent [31–33], the mechanism remains largely unknown. Therefore, it should be an interesting topic to investigate why the expression of TRPC6 is cell cycle-dependent and whether such dependence is unique in cancer cells. In other words, enhanced TRPC6 expression may be critical for lung cancer cell cycle progression. However, it remains unknown how TRPC6 expression is elevated in the cells at the S-G2/M phase.

Our data show that inhibition of TRPC6 with its blocker or siRNA caused A549 cell detached, but did not induce any types of cell death, because after replating the detached cells into dishes in the absence of the blocker or siRNA of TRPC6, these detached cells can get attached again and grow back to form a cell monolayer (Figure 5). We have also found that inhibition of TRPC6 activity or expression can reduce the expression of an adherent molecule, fibronectin, and a tight junction protein, ZO-1 (Figure 6D, 6E). Since it is known that TRPC6 is a Ca^2+^-permeable channel [21] and that intracellular Ca^2+^ modulates the expression of fibronectin and ZO-1 [34; 35], we argue that intracellular Ca^2+^ plays an important role in mediating TRPC6-regulated expression of fibronectin and ZO-1. Therefore, inhibition of TRPC6, at least within 24 h, only abolishes the adherent ability of this NSCLC cell line possibly by reducing the expression of fibronectin, and ZO-1. Fibronectin is a glycoprotein in extracellular matrix which promotes lung cancer cell migration and invasion [36; 37]. Together with fibrin, fibronectin can form a provisional wound matrix around the tumor nodules to provide a favorable substrate for cell migration and invasion [38]. Fibronectin may regulate tumor cell migration, invasion, and metastasis also by interacting with integrins [39]. Not only fibronectin, ZO-1 may also promote cancer cell migration because silencing of ZO-1 can reduce directional mobility [40]. The present study, for the first time, show that inhibition of TRPC6 reduces the invasion of A549 lung cancer cells possibly associated with decreased expression of fibronectin and ZO-1. Therefore, investigation of the role of fibronectin and ZO-1 in mediating TRPC6 regulation of cancer cell invasion may serve as an interesting topic for our future studies.

Since TRPC6 knockout fails to produce any severe phenotype in mice [41], we argue that unlike other TRP channels, TRPC6 appears to be less important for normal cell function. Therefore, activated TRPC6 may be a unique hallmark in malignant cells, and according to the TRPC6 knockout mouse model, targeting of TRPC6 should not affect normal cell function and thus is feasible for treatment of cancers. In conclusion, the present study provides strong evidence at the molecular level for supporting the clinical trials focused on the use of TRPC6 blockers in cancer therapy.

## MATERIALS AND METHODS

### Cell culture and TRPC6 knockdown

A549, a human lung adenocarcinoma epithelia cell line, was purchased from American Type Culture Collection. A549 cells were cultured in RPMI 1640 medium containing 10% fetal bovine serum, 100 U/mL penicillin, and 100 μg/mL streptomycin. Cells were cultured in a humidified 5% CO_2_ incubator at 37°C, and the medium was changed every other day. To knockdown TRPC6 expression, A549 cells were transiently transfected with siRNA directed against TRPC6 (80 nM, Santa Cruz Biotechnology, Cat#: SC-42673) according to the protocol provided by the manufacture. Cells transfected with control (scrambled) siRNAs (80 nM, Santa Cruz Biotechnology, Cat#: SC-37007) served as a control. All the experiments using these cells were carried out within 72 h after the transfections. The reduction of TRPC6 expression was confirmed by Western blot experiments. All the experiments in this study were performed at room temperature.

### Cell proliferation assays

Cell proliferation was evaluated by performing CellTiter 96^®^ AQ_ueous_ One Solution Cell Proliferation Assay (MTS). MTS assay was carried out according to the manufacturer's instruction. Briefly, A549 cells, either under control conditions or after experimental manipulations (as described in the Results), were transferred into 96 well plates. 10 μl of CellTiter 96^®^ AQ_ueous_ One Solution was added into each well. After the cells were incubated at 37°C for 3 h, the absorbance was detected at 490 nm with the Synergy 4 Microplate Reader (BioTek). Three independent experiments were performed for each experimental condition.

### Western blot

Either control A549 cells or treated cells were cultured as described above. Cell lysates (100 μg) were loaded and electrophoresed on 7.5% SDS-PAGE for 60 to 90 min. Gels were transferred onto polyvinylidene fluoride (PVDF) membranes for 60 min at 90 volts. After 1 hour blocking with 5% nonfat milk-PBST buffer, the PVDF membranes were incubated overnight at 4°C with primary antibody (1:1000 dilution) of rabbit polyclonal antibody to TRPC6 (Alomone Labs, Cat#:ACC-017), rabbit polyclonal antibody to Fibronectin (Sigma, Lot#: F3648), mouse polyclonal antibody to ZO-1 (Invitrogen), or rabbit polyclonal antibody to β-actin (1:2500) (Santa Cruz Biotechnology, Lot#: D2409). After 3 vigorous washes, the membrane was then incubated for 1 h with either horseradish peroxidase (HRP)-conjugated donkey anti-rabbit IgG secondary antibody (1:5000 dilution, GE healthcare, Lot#:NA934V) or HRP-linked anti-mouse IgG secondary antibody (1:5000 dilution, BD Transduction Laboratories™). Finally, blots were visualized with chemiluminescence using ECL Plus Western Blotting Detection System (GE healthcare).

### Confocal microscopy imaging

Cycle analysis was carried out using the fluorescent ubiquintin-based cell cycle indicator (Fucci^®^), as described previously [42]. A549 cells were transiently transfected with Premo™ FUCCI Cell Cycle Sensor (Molecular Probes, Cat#: P36237) according to the protocol provided by the manufacturer. Premo™ FUCCI contains Premo™ geminin-GFP to label cells in G2/M phases shown in green and Premo™ Cdt-RFP to label G1/S phases shown in red. Briefly, after plating A549 cells at a desired density and allow sufficient time for cells to adhere, the Premo™ reagent with the volume 40-80 particles per cell was added to the cells under control or treated conditions (as described in the Results). The cells were returned to the culture incubator overnight. After 16 to 24 hours, the cells were fixed with 4% paraformaldehyde for 10 min and permeabilized with 0.1% Triton X-100 for in NaCl bath solution 10 min. The cells were incubated with rabbit polyclonal antibodies to TRPC6 (Alomone Labs, Cat#:ACC-017) for 1 h, washed twice, incubated with a secondary antibody (Alexa Fluor^®^ 405-tagged goat anti-rabbit IgG, Molecular Probes, Lot: 1476593) at room temperature for 1 h. To determine intracellular Ca^2+^, A549 cells were incubated with 5 μM fluo-3 AM, a fluorescent Ca^2+^ indicator, for 30 min in the incubator. In each set of experiments, each wash was very gentle in order to avoid floating the detached cells, and the images were taken using the same parameter settings. After the cells were labeled with Fucci, confocal microscopy XY scanning was performed within 5-15min and used 405 nm, 488 nm, or 543 nm laser to respectively excite the indicators. The emissions at 463 nm (for TRPC6), 519 nm (for either cells at the S-G2/M phase or intracellular Ca^2+^), or 603 nm (for cells at the G1 phase) were measured and used for imaging analysis. 3-D images were reconstructed according to the Z stacks.

### Patch-clamp technique

The whole-cell recordings were performed as we described previously [43]. Briefly, before electrophysiological analysis, A549 cells on the petri dish were washed with NaCl bath solution (see Solutions) and mounted on the stage of a Nikon inverted microscope. Polished patch pipettes of borosilicate glass typically with about 5 MΩ were used for patch-clamp recording. Patch pipettes were filled with NaCl pipette solution (see Solutions). Only patches with high resistance seals (above 5 GΩ) were used in the experiment to form the whole-cell configuration. Whole-cell currents were recorded using an Axopatch-200B amplifier and pClamp 10 software (Molecular Devices) and low-pass filtered at 2 kHz. A voltage-ramp protocol from −80 to 80 mV was used to quickly get the current-voltage relationship; the protocol was given at an interval of 1 min. All the experiments were performed at 22-23°C.

### Invasion assays

Cell invasion assay was performed using 6.4 mm Biocoated Transwell inserts (8 μm pore size, BD, Cat#:354480), according to the protocol provided by the manufacturer, as previously reported [44]. Briefly, the chambers were added warm culture medium to the interior of the inserts and bottom of wells. After 2 h rehydrating in a humidified tissue culture incubator, the medium was carefully removed without disturbing the layer of Matrigel™ Matrix on the membrane. A549 cells were seeded at 50,000 cells per well into Matrigel-coated trans-well chambers. After the cells were attached, experimental manipulations were performed. The non-invading cells were quickly removed from the membrane surface where the cells were plated with cotton tipped swabs. The cells passed through the 8 μm pores on to the other surface of the membrane were fixed with 100% methanol for 15 min and then stained with Eosin for 15 min. The membrane was excised from the insert and mounted on a microscope slide. The images of cells that passed through the membrane were examined under a microscope. The number of cells was counted and averaged per microscopy field.

### Solutions

The NaCl bath solution contained (in mM): 145 NaCl, 5 KCl, 1 CaCl_2_, 1 MgCl_2_, and 10 HEPES, adjusted pH to 7.4 with NaOH. The NaCl pipette solution contained (in mM): 140 NaCl, 5 KCl, 1 MgCl_2_, 2 ATP-Na_2_, 10 HEPES and 50 nM (1 μM) free Ca^2+^ (after titration with 2 mM EGTA), adjusted to pH 7.2 with NaOH.

### Statistical analysis

Results are shown as means ± SD. Student's *t*-test was used for comparison between two groups. One-way analysis of variance was applied for comparison among multiple groups. Two-way analysis of variance was applied for comparison among multiple groups with two subgroups. Differences with a *P* value of < 0.05 were considered statistically significant.
